# Identification and characterization of the LRR repeats in plant LRR-RLKs

**DOI:** 10.1186/s12860-021-00344-y

**Published:** 2021-01-28

**Authors:** Tianshu Chen

**Affiliations:** State Key Laboratory of Pharmaceutical Biotechnology, School of Life Sciences, Nanjing University, 163 Xianlin Ave, Nanjing, 210046 China

**Keywords:** Plant LRR-RLKs, N-glycosylation, Ligand binding, LRR motif prediction, PSSM

## Abstract

**Background:**

Leucine-rich-repeat receptor-like kinases (LRR-RLKs) play central roles in sensing various signals to regulate plant development and environmental responses. The extracellular domains (ECDs) of plant LRR-RLKs contain LRR motifs, consisting of highly conserved residues and variable residues, and are responsible for ligand perception as a receptor or co-receptor. However, there are few comprehensive studies on the ECDs of LRR-RLKs due to the difficulty in effectively identifying the divergent LRR repeats.

**Results:**

In the current study, an efficient LRR motif prediction program, the “Phyto-LRR prediction” program, was developed based on the position-specific scoring matrix algorithm (PSSM) with some optimizations. This program was trained by 16-residue plant-specific LRR-highly conserved segments (HCS) from LRR-RLKs of 17 represented land plant species and a database containing more than 55,000 predicted LRRs based on this program was constructed. Both the prediction tool and database are freely available at http://phytolrr.com/ for website usage and at http://github.com/phytolrr for local usage. The LRR-RLKs were classified into 18 subgroups (SGs) according to the maximum-likelihood phylogenetic analysis of kinase domains (KDs) of the sequences. Based on the database and the SGs, the characteristics of the LRR motifs in the ECDs of the LRR-RLKs were examined, such as the arrangement of the LRRs, the solvent accessibility, the variable residues, and the N-glycosylation sites, revealing a comprehensive profile of the plant LRR-RLK ectodomains.

**Conclusion:**

The “Phyto-LRR prediction” program is effective in predicting the LRR segments in plant LRR-RLKs, which, together with the database, will facilitate the exploration of plant LRR-RLKs functions. Based on the database, comprehensive sequential characteristics of the plant LRR-RLK ectodomains were profiled and analyzed.

**Supplementary Information:**

The online version contains supplementary material available at 10.1186/s12860-021-00344-y.

## Background

To adapt to sessile lifestyles, plants need to sense various signals from the outside world in response to various environmental changes. Some plants have evolved to meet this challenge by receiving these signals via cellular membrane-localized receptor-like kinases (RLKs) [1–4]. The largest family of such receptors is termed leucine-rich-repeat (LRR) RLKs, which are involved in multiple developmental processes as well as disease resistances [4, 5]. LRR-RLKs are composed of an extracellular domain (ECD), which is responsible for ligand binding, a single membrane-spanning helix (TM), and a cytoplasmic kinase domain (KD) [4]. Typically, the plant LRR-RLK family is classified into 15–20 subgroups (SGs) based on phylogenetic analysis of the KDs and is denoted according to the SGs in Arabidopsis (*Arabidopsis thaliana*) LRR-RLKs (numbered with Roman numerals) [2, 6–9]. Although the classification of the LRR-RLK genes tends to rely on the phylogenetic analysis of the KDs due to the ambiguous alignment of the ECDs, similar structural arrangement patterns of the ECDs are often observed in most SGs [10]. In addition, in flowering plants, a more extensive selection pressure is imposed on ECDs than on KDs or TM in order to adapt to more sophisticated ligands recognition [5, 7, 11, 12].

LRRs share a common structure of 20–43 continuous residues uncommonly rich in the hydrophobic amino acid leucine [13]. Seven distinct LRR subfamilies have been identified, where the LRRs in LRR-RLKs share plant-specific consensus sequences (CS) such as LxxLxLxxNxL(s/t) GxLPxxLxxLxx (“L” refers to a hydrophobic amino acid, “N” refers to an asparagine, threonine, serine or cysteine, and “x” refers to variable residue) [14, 15]. Recently resolved structures reveal that the highly conserved region “LxxLxLxxN” in LRRs tend to assemble into a curved parallel β-sheet lining the inner circumference of their solenoid structure and the highly conserved region “L(s/t)GxLP” formed the plant-specific second β-strand which forced the LRR stacks out of a plain and into a rod, curve, and eventually superhelical assembly [14–16]. Therefore, the 16-residue segment “LxxLxLxxNxL(s/t)GxLP” could be taken as the plant-specific highly conserved segment (HCS). Moreover, according to the reported LRR-RLK-ligand complexes, the residues of the inner side of the ECDs are crucial for proper functioning of LRR-RLKs, as the inner surfaces bound the ligands to supply a platform for recruiting co-receptors to activate a signaling pathway in a structure complementary way [16–18]. Moreover, plant LRR-RLKs usually harbor heavy N-glycosylation modifications, which tend to located at the canonical asparagine-linked (Asn/N-) glycosylation sites, NxS/T sequons (x ≠ P) [19–21]. The N-glycosylation modifications are believed to contribute to the proper folding, trafficking and biological functioning of LRR-RLKs [19, 21–23]. Therefore, the efficient prediction of the extracellular LRR motifs and sequentially comprehensive analysis of ECDs for plant LRR-RLKs will benefit their functional characterization and binding site analysis.

To predict LRR regions, many methods are available that are based on the hidden Markov model (HMM) or sequence alignment with previously known LRRs, such as SMART [13], Pfam [24], which cannot effectively predict the most divergent LRRs in a given sequence [25]. Recently, two methods, LRRfinder [26] and LRRsearch [25], were conducted based on the position-specific scoring matrix algorithm (PSSM); these methods were proved to be powerful tools in predicting LRR motifs. The fact that LRRfinder performed well in Toll-like receptors (TLRs), whereas LRRsearch performed well in cytoplasmic NOD-like receptors (NLRs) [25] indicates that the efficiency of the PSSM based method strongly relies on the training datasets.

In the current study, I developed the “Phyto-LRR prediction” program to identify plant-specific LRR motifs with high efficiency using the PSSM algorithm, which was trained by the plant-specific 16-residue LRR-HCSs with some optimizations. Based on this program, more than 55,000 LRR motifs from 3987 protein sequences with LRRs, TM and KDs from 17 represented fully sequenced land-plant genomes were detected and stored in the database. Those with signal peptides were then classified into 18 SGs according to the maximum-likelihood phylogenetic analysis of the KD sequences and the LRRs arrangement in the ECDs was determined. Different from the remaining SGs, SG_x had two clusters of LRR numbers and density in the ECDs, which were then denoted as SG_x_1* and SG_x_2*, respectively, based on further phylogenetic analysis of SG_x. According to the database, some characteristics of the LRR motifs in each SG of the LRR-RLKs were examined, such as the density of the LRRs, the solvent accessibility, the variable residues, and the N-glycosylation sites, revealing a comprehensive profile of the plant LRR-RLK ECDs.

## Results

### The construction of the Phyto-LRR database

**Fig. 1:**
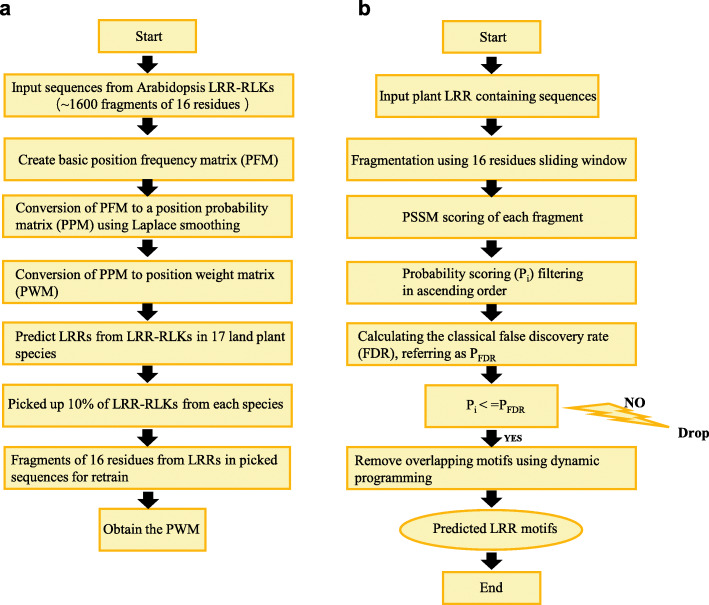
The flow chart of the Phyto-LRR-prediction program. **a** The process of training the PSSM matrix. **b** Flow chart describing the prediction process of the program

In total, 3987 protein sequences containing LRR(s), TM, and a KD from 17 represented embryophyte genomes were extracted for LRR motif prediction (see Methods), including four monocot genomes, ten dicot genomes, the liverwort *Marchantia polomorpha*, the moss *Physcomitrella patens*, and the spikemoss *Selaginella moellendorffii*. The species names, five-digit codes and the number of sequences extracted are listed in Table 1. From the ECD sequences of these protein sequences, 55,457 LRR motifs were predicted by the Phyto-LRR prediction program (Fig. 1; see Methods) and saved in the Phyto-LRR database (both the prediction tool and the database can be freely accessed at http://phytolrr.com for website usage and at http://github.com/phytolrr for local usage). The accuracy of this program was determined by comparing its predicted outcomes with LRRs identified in the crystal structures. As shown in Table 2, the Phyto-LRR prediction program performed better than SMART and Pfam, which are available online tools based on HMM and/or sequence alignment. For LRR-RLK/RLP sequences in the training dataset, the accuracy of the Phyto-LRR prediction program reached 99%, and for those in the independent test dataset, the accuracy was about 92%, indicating that this program could identify LRR motifs in plant LRR-RLKs/RLPs with high efficiency. Since there are only tens of the LRR- RLK/RLP sequences with crystal structures available online, the diversity of the current independent test dataset is restrained. To further assess the performance of this program, two LRR-RLK sequences were randomly picked from each of the 17 species, and the LRRs of the ECDs were predicted by other two available PSSM based programs, the LRRfinder and the LRRsearch (Tab. S1). The results showed that the Phyto-LRR prediction program could identify LRR motifs in plant LRR-RLK proteins the most efficiently compared with the other tools. When predicting LRR motifs, Phyto-LRR sometimes missed very divergent motifs located at the N terminal and/or the C terminal, such as the SOBIR1 and PGIP in Table 2, therefore, the database obtained was manually checked (Tab. S2), especially for LRRs in the N terminal and/or the C terminal, before it was employed for further analysis. Despite the predicted LRR motif offsets, the database was also integrated with the prediction of the secondary structures, the soluble accessibility, as well as potential canonical N-glycosylation sites [NxS/T (x ≠ P)] (see Methods).

**Table 1:** The number of LRR-RLK protein sequences in 17 represented land plants in the Phyto-LRR prediction database

Species	Five Digit Code	Seq Num.
*Amborella trichopoda*	AMBTR	108
*Arabidopsis lyrata*	ARALY	207
*Arabidopsis thaliana*	ARATH	213
*Brachypodium distachyon*	BRADI	222
*Brassica rapa*	BRARA	273
*Glycine max*	GLYMA	424
*Marchantia polymorpha*	MARPL	102
*Medicago truncatula*	MEDTR	316
*Oryza sativa ssp. indica*	ORYSI	287
*Oryza sativa ssp. japonica*	ORYSJ	281
*Phoenix dactylifera*	PHODC	149
*Physcomitrella patens*	PHYPA	162
*Populus trichocarpa*	POPTR	373
*Selaginella moellendorffii*	SELML	138
*Solanum lycopersicum*	SOLLC	213
*Solanum tuberosum*	SOLTU	291
*Zea mays*	MAIZE	228
Total		3987

**Table 2:** The performance of the Phyto-LRR prediction program. Sequences from the PDB files were used to examine the performance of the LRR prediction program

Protein Name	Species	Uniprot ID	PDB code	Gene accession	Phyto-LRR (Accuracy)	SMART (Accuracy)	Pfam (Accuracy)
**Training dataset of plant LRR-RLK/RLP with crystal structures**							
RGFR1	*Arabidopsis thaliana*	C0LGR3	5hz1	At4g26540	24 (96%)	8 (33%)	10 (42%)
PXY/TDR	*Arabidopsis thaliana*	Q9FII5	5jfk	At5g61480	23 (96%)	9 (39%)	10 (43%)
BRI1	*Arabidopsis thaliana*	O22476	3rgz	At4g39400	25 (100%)	7 (28%)	13 (52%)
BRL1	*Arabidopsis thaliana*	Q9ZWC8	4j0m	At1g55610	24 (100%)	8 (33%)	12 (50%)
PSKR1	*Arabidopsis thaliana*	Q9ZVR7	4z63	At2g02220	21 (100%)	6 (29%)	11 (52%)
PEPR1	*Arabidopsis thaliana*	Q9SSL9	5gr8	At1g73080	27 (100%)	9 (33%)	13 (48%)
FLS2	*Arabidopsis thaliana*	Q9FL28	4mna	At5g46330	24 (100%)	13 (54%)	13 (54%)
HAE	*Arabidopsis thaliana*	P47735	5ixo	At4g28490	22 (100%)	8 (36%)	11 (50%)
**Average accuracy**					99%	36%	45.5%
**Test dataset of plant LRR-RLK/RLP with crystal structures**							
SOBIR1	*Arabidopsis thaliana*	Q9SKB2	6r1h	At2g31880	6 (67%)	0 (0%)	0 (0%)
PRK6	*Arabidopsis thaliana*	Q3E991	5yah	At5g20690	6 (100%)	4 (67%)	4 (67%)
SERK2	*Arabidopsis thaliana*	Q9XIC7	6g3w	At1g34210	5 (80%)	0 (0%)	3 (60%)
ERL1	*Arabidopsis thaliana*	C0LGW6	5xjo	At5g62230	20 (100%)	10 (50%)	11 (55%)
TMM	*Arabidopsis thaliana*	Q9SSD1	5xjo	At1g80080	10 (91%)	5 (50%)	5 (50%)
TMK1	*Arabidopsis thaliana*	P43298	4hq1	At1g66150	13 (91%)	5 (38%)	8 (62%)
BIR2	*Arabidopsis thaliana*	Q9LSI9	6fg7	At3g28450	5 (100%)	0 (0%)	4 (80%)
ERL2	*Arabidopsis thaliana*	Q6XAT2	5xkn	At5g07180	20 (100%)	9 (45%)	10 (50%)
PGIP	*Phaseolus vulgaris*	P58822	1ogq		9 (90%)	0 (0%)	5 (55%)
TMK3	*Arabidopsis thaliana*	Q9SIT1	7brc	At2g01820	13 (92%)	5 (38%)	7 (54%)
ERL2	*Arabidopsis thaliana*	Q6XAT2	5xkn	At5g07180	20 (100%)	9 (45%)	10 (50%)
PRK3	*Arabidopsis thaliana*	Q9M1L7	5wls	At3g42880	6 (100%)	0 (0%)	4 (67%)
**Average accuracy**					92.6%	28%	54%

### The distribution of LRR-RLKs in different LRR-RLK subgroups of different species

**Fig. 2:**
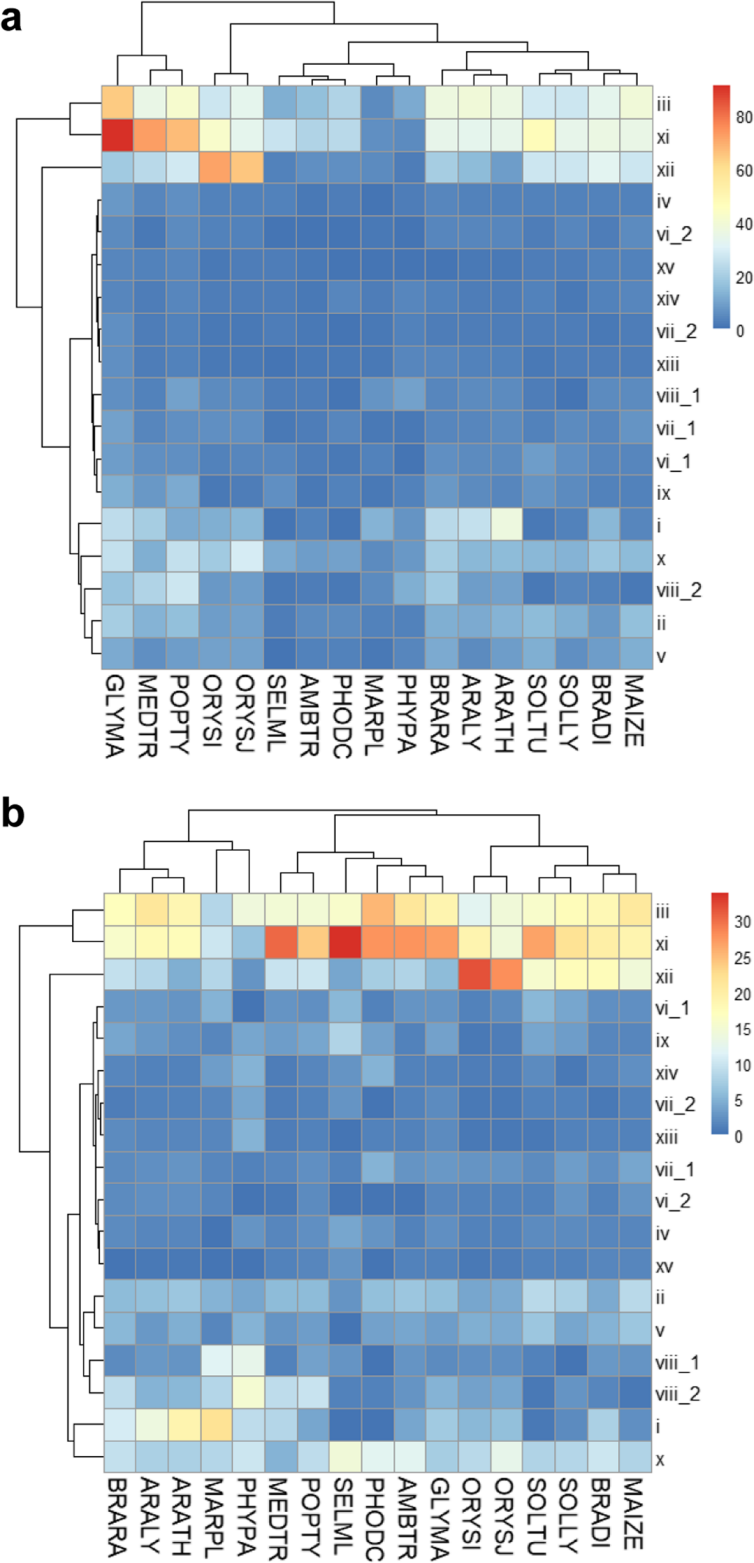
The distribution of potential LRR-RLK sequences from different species in different subgroups (SGs). **a** The number of LRR-RLK candidates in different SGs from different species. **b** The percentage of LRR-RLK candidates in different SGs of each species. The global perspective of the sequences in each SG was presented as a heatmap using the R package pheatmap [27]

In total, 2999 protein sequences from the Phyto-LRR database containing a signal peptide, LRR(s), TM, and a KD were classified into 18 SGs based on the maximum likelihood (ML) phylogenic analysis of the KD sequences in IQtree (Table 3; Fig. S1, Tab. S3; See Methods). To test the robustness of the ML tree, ten subsets containing about 300 sequences consisting of ~ 10% random sequences from each SG noted in the global phylogenetic tree were selected (Tab. S3) to construct the test ML phylogenic trees. The results showed that the SGs were mainly monophyletic in most test trees except that SG_x, SG_xi, and SG_xii, showing one to three sequence were placed outside the main monophyletic clade in more than five test trees. The distribution features of the LRR-RLK sequences were then examined. According to Fig. 2, SG_iii, SG_xi, and SG_xii had a larger number and percentage of LRR-RLK sequences than other SGs in all 17 species. By contrast, although ancient species, such as PHYPA, MARPL, PHODC AMBTR, and SELML, contained fewer sequences in each SG (Fig. 2a), the distribution pattern of the sequences in each SG were not significantly different from other species in most SGs (Fig. 2b). Interestingly, in the ancient species and the GLYMA, the sequences accounted for a much higher percentage in SG_xi than in other species (Fig. 2b).

**Table 3:** The number of LRR-RLK protein sequences for phylogenetic analysis and the average number of LRR repeats in the extracellular domain (ECD) of these genes for each subgroup (SG) among 17 species

Subgroup	Seq Num.	Average LRR Num. of the ECD
i	211	3.58
ii	173	4.91
iii	504	8.09
iv	54	6.81
v	121	6.58
vi_1	77	9.89
vi_2	43	5.20
vii_1	73	18.71
vii_2	33	26.54
viii_1	76	11.41
viii_2	150	9.77
ix	83	11.89
x_1*	117	8.04
x_2*	145	24.82
xi	639	22.41
xii	385	21.49
xiii	37	5.09
xiv	45	9.33
xv	29	16.65
Total	2995

### The LRR motif arrangement in different LRR-RLK SGs

**Fig. 3:**
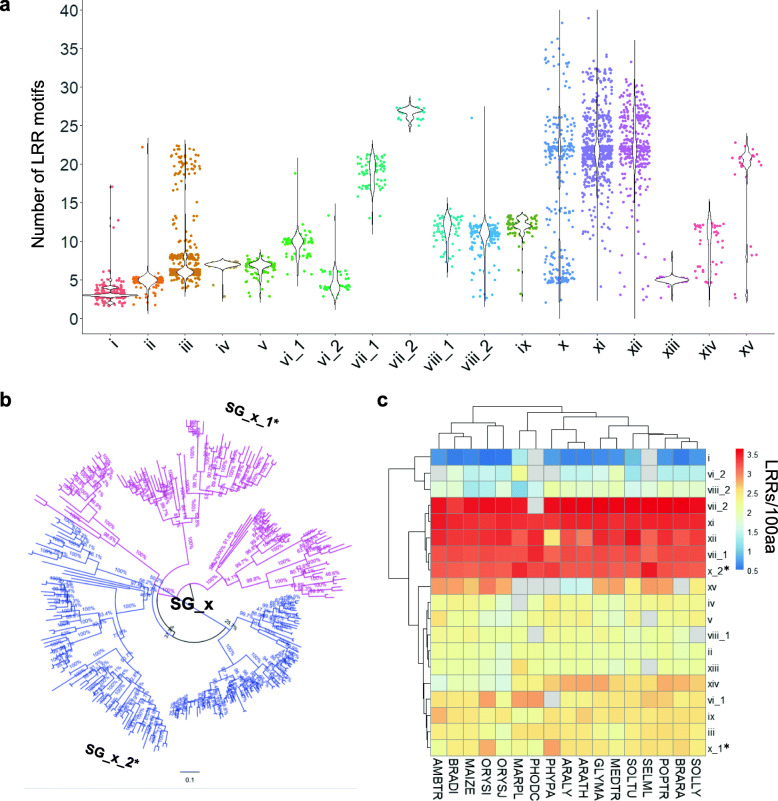
LRR motif distribution pattern in plant LRR-RLKs. **a** The number of predicted LRR motifs in different SGs. **b** SG_x_1* and SG_x_2* were clustered in the refined phylogenetic tree. Refined phylogenetic tree of SG_x was constructed based on the kinase domains (KDs) using the neighbor-joining (NJ) method within MEGA software (version 7.0.26). The bar indicated a mutation rate of 0.10 substitutions per site. Bootstrap values of 1000 replications were shown near the branch. **c** LRR motifs distribution of the plant LRR-RLK ECDs from different species in different SGs. LRR motifs per 100 amino acids of the ECDs were calculated and shown as a heatmap using the R package pheatmap [27]

Based on the phylogenetic analysis and the Phyto-LRR database, the distribution pattern of the LRRs in the ECDs was determined. According to peaks of each violin plot in Fig. 3a, for most SGs, the probability densities of the LRR numbers in each sequence were high in a small interval with a small percentage of outliers except for SG_x, which held two peaks (almost equal) of the LRR number, concentrated around 4–10 and 18–22, respectively, implying that there were two distinct ECDs in the SG_x and that these two subclades in SG_x should function in different ways. Since, these two subclades intersected with each other in the big tree, further phylogenetic analysis of the KDs of SG_x were conducted. The results turned out that these two subclades in SG_x were clustered separately, therefore, in this work, the SG_x was further divided into SG_x_1* and SG_x_2* (Fig. 3b). The density of the LRR motifs in the ECDs was also calculated, which is described as the number of LRRs per 100 amino acids. As shown in Fig. 3c, SG_i had the lowest LRR density, which was less than 0.6 LRRs per 100 amino acids, implying that only a few LRRs existed in the ECDs of the SG_i members. By contrast, in SG_vii, SG_x_2*, SG_xi, and SG_xii, LRR motifs exceedes 3.3 per 100 residues, suggesting that LRR motifs appeared continuously with few insertion sequences in these SGs. In agreement with Fig. 3a, the two sub-clusters in SG_x showed distinct LRR densities (Fig. 3c).

### The features of the residues located on the inner side of the superhelical LRR assemblies

**Fig. 4:**
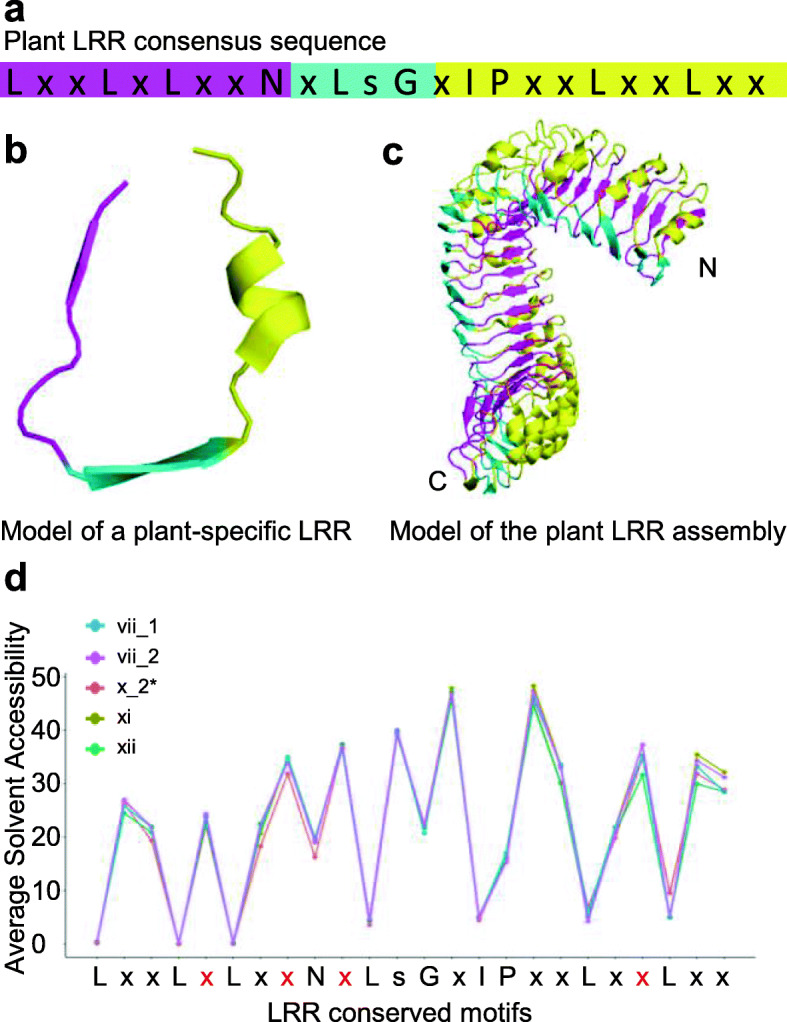
The LRR motif signatures in plant LRR-RLKs SG_vii, SG_x, SG_xi and SG_xii with more than 20 LRR repeats. **a** The consensus sequences of the LRR motif. **b** The model of a plant LRR unit. **c** The model of LRR stacking of continuous LRR repeats. Typical extracellular LRR architectures (based on PDB code 3RGZ) are shown. The LxxLxLxxN motifs were taken as the internal side of the superhelix, the xLsG motifs were taken as the lateral of the superhelix, and the xIPxxLxxLxx motifs were taken as the external side of the superhelix. The motifs are shown in magenta, cyan, and yellow, respectively. **d** The average solvent accessibility of LRR motifs. The solvent accessibility of the LRR-RLKs extracellular domains was predicted in the SCRACH-1D program [31]. The average solvent accessibility value of residues in LRR motifs predicted by ACCpro20 is shown

In plants, for sequences with tens of continuous LRRs and few insertion segments, the ectodomain of the LRR-RLKs tended to stack into superhelical shapes. Such superhelical ECDs tend to act as receptors to sense various ligands for signal activation [28–30]. The structures of the LRR assemblies are predictable due to the high conservation of the LRR repeats, with the “LxxLxLxxN” forming the inner side of the superhelix, the “xLs/tG” forming the plant-specific second β-sheet on the lateral side, and the remainder forming the backside (Fig. 4a-c). Since proteins tend to bury their hydrophobic patches inside during the folding process, the prediction of the solvent accessibility of the LRR motifs in such LRR stacks may assist in better understanding their important structural elements. Plant LRR-RLKs in SG_vii, SG_x_2*, SG_xi, and SG_xii had fewer sequential insertions (Fig. 3c) and tended to form superhelical structures with more than 20 LRRs. Therefore, sequences of this type were selected (Fig. 3a), and the average solvent accessibility scores of each SG were predicted by ACCpro20 [31] (Fig. 4d). The results showed that the conserved residues on the LRR backbone were more hydrophobic than variable residues, even for the hydrophilic residues, such as the conserved asparagine (9th) and glycine (13th), with “L” sites the most hydrophobic (Fig. 4d). Moreover, variable residues at the 3rd, 7th, 18th, and 20th sites had lower hydrophilicity than other variable residues (Fig. 4d), which might, to some extent, be more important in protein proper folding.

**Fig. 5:**
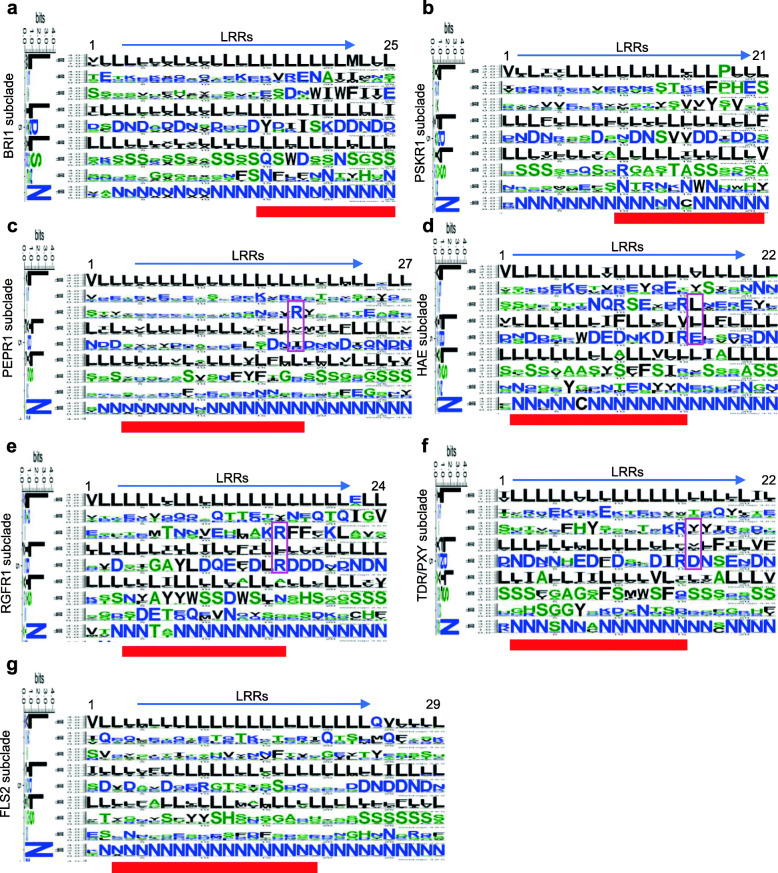
The sequential characteristics of the variable residues in the inside of the superhelix. The footprints of residues along the inner side of the LRR stacks in seven homolog clades (Fig. S2 and S3) are shown. Red bars indicated LRR areas involved in ligand binding according to LRR-ligand complexes: BRI1 (PDB 4M7E), PSKR1(PDB 4Z61), PEPR1 (PDB 5GR8), HAE (PDB 5IXQ), RGFR (PDB 5Z21), TDR/PXY (PDB 5GIJ), and FLS2 (PDB 4MN8). The conserved RxR motifs in sequences belonging to SG_xi are highlighted in magenta boxes

The evolutional analysis and structural analysis revealed that variable residues located on the inner side of the superhelical stacks were crucial for LRR-RLKs ligand perception [7, 16]. Based on the Phyto-LRR database, the residues could be comprehensively profiled in both sequential and spatial dimensions, i.e. both the sequential conservation of the residues and their special localization at the superhelix could be displayed, which will assist in finding important functional residues of the LRR-RLKs with convincing homolog models. Here, the author profiled the residues located at the inner side of the superhelical stacks in several homolog clusters containing well-studied LRR-RLKs. The Arabidopsis BRI1 (AT4g39400), PEPR1 (At1g73080), FLS2 (At5g46330), HAE (At4g28490), TDR/PXY (At5g61480), PSKR1 (At2g02220), and RGFR1 (At4g26540) protein sequences were used as queries to perform BLASTP search for their own homologous sequences in the LRR-RLK sequences in the global ML tree (Table 3; Fig. S1; Tab. S3). For each group of sequences, protein sequences of the top 300 hits were selected and 10 hits (or all hits if the total number was less than 10) in each genome were chosen (Tab. S9) for phylogenetic analysis. The BRI1, PEPR1, FLS2, HAE, TDR/PXY, PSKR1, and RGFR1 subclades were extracted for further residue analysis (Fig. S2). For each homolog subclade, the logo of the LRRs and the logos of the residues at each site of each LRR were created (Fig. S3), so that the residues conservation at each site could be observed (Fig. 5). In this work, The BRI1-subclade and PSKR1-subclades belonged to SG_x_2*, the FLS2-subclade belonged to SG_xii, and the remainder originated from SG_xi. In each subclade, residue logos at each site of each LRR were created. According to Fig. 5, although the variable residues at the 2nd, 3rd, 5th, 6th, and 7th sites in the LRR backbone were distinct among clades, in close homolog subclades, they were highly conserved in certain positions. Some of the highly conserved residues have been well-documented, such as the RxR motif in AtPEPR1, AtHAE, AtGRFR1, and AtPXY (Fig. 5c-f, motifs denoted in magenta boxes), others remain less known. Since these highly conserved residues appeared at ligand binding areas in their well-studied Arabidopsis homologs (Fig. 5, LRRs highlighted in red bars), these residues might be involved in ligand recognition. Moreover, residues lying on the 5th and 7th sites in each LRR are often exhibited DLS or NLS motifs, and such residues pairs tended to appear in LRRs not involved in ligand binding, indicating that these DLS or NLS motifs might contribute to protein structural integrity.

### The distribution of the N-glycosylation sites in the LRR-RLK ECDs

**Fig. 6:**
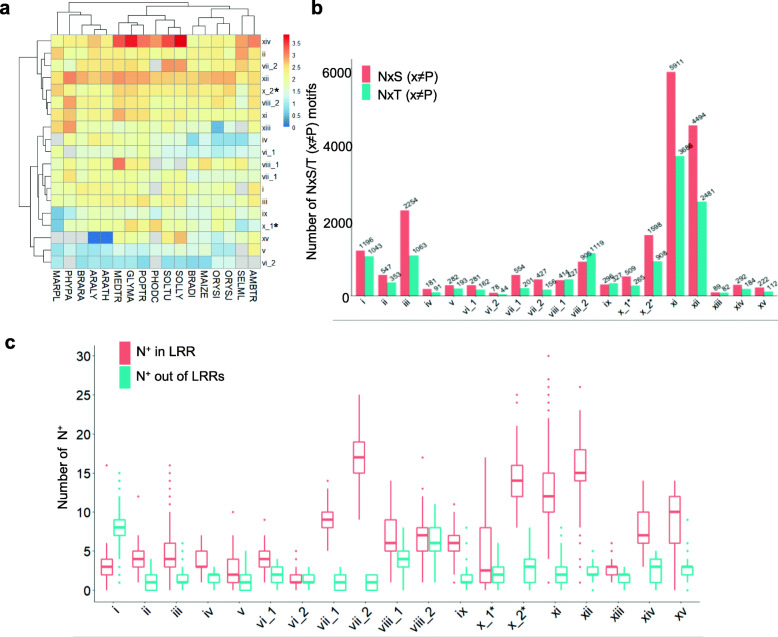
The distribution of N^+^ of the plant LRR-RLKs extracellular domains. **a** N^+^ distribution of the plant LRR-RLK extracellular domains from different species in different SGs. The N sites in NxS/T (x ≠ P) are noted as N^+^ for short. N^+^ per 100 amino acids of the ECDs was calculated and showed as a heatmap using the R package pheatmap [27]. **b** The types of N^+^ in each SG. The number of NxS (x ≠ P) and NxT (x ≠ P) motifs in different SGs was determined. **c** N^+^ distribution of the plant LRR-RLKs ECDs internal or external to LRR motifs. N^+^ locations in the range from the start point of the indicted LRR motifs to the start point of the LRR motifs plus 24 were considered as N-glycosylation sites located in LRRs

Another important feature of plant LRR-RLK ECDs is that they are heavily N-glycosylated. According to the analysis of the Arabidopsis manually annotated proteome GFF file from Swiss-Prot, among the 156 identified LRR-RLKs, more than 80% of sequences harbored an average of more than 5 N-glycosylation sites and 98% of the N-glycosylation sites were annotated to be N-glycosylated (Tab. S4), implying that N-glycosylation is an important modification and could be predicted based on the NxS/T (x ≠ P) sequons. Here, N sites on the NxS/T (x ≠ P) sequons were denoted as N^+^ for short and their distribution features were illustrated. At first, the number of N^+^ per 100 amino acids of the ECDs were determined in each SG from different species. As shown in Fig. 6a, no apparent clusters of N^+^ density differences could be observed among different plant species. The number of N^+^ was rich in SG_ii, SG_vii_2, SG_viii_2, SG_x_2*, SG_xi, SG_xii and SG_xiv, especially in SG_xiv from MEDTR, GLYMA, POPTR, SOLTU, PHODC and SOLLY. By contrast, in SG_v and SG_vi_2, the N^+^ density was lower than that in other SGs. The ratio between NxS and NxT was also examined, and it showed that plant LRR-RLKs had a preference for NxS sequons in all SGs except SG_viii_1 and SG_ix (Fig. 6b). N^+^ sites were mainly localized on LRR repeats in most SGs, especially for SG_vii, SG_x_2*, SG_xi, and SG_xii, whereas they were mainly not localized on LRR repeats in SG_i (Fig. 6c) due to its lower LRR concentration of ECD (Fig. 3c).

**Fig. 7:**
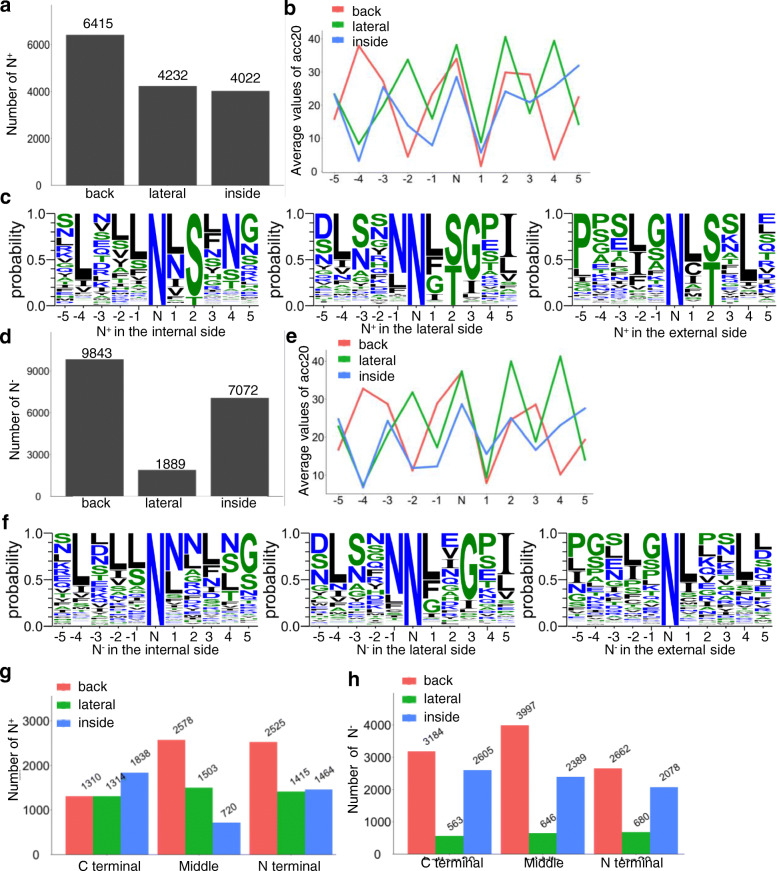
The signatures of N^+^ and N^−^ in SG_vii, SG_x, SG_xi, and SG_xii with more than 20 LRRs. **a** and **d** The total number of N^+^ and N^−^ the internal, lateral, and external areas of the plant LRR-RLK ectodomain. **b** and **e** The solvent accessibility of the residues next to N^+^ and N^−^ on the internal, lateral, and external side of the plant LRR-RLK ectodomain superhelix. The ACCpro20 values were calculated, and the average ACCpro20 values of residues located −5 to + 5 from the N sites are shown. **c** and **f** The motif signatures of N^+^ and N^−^ on the internal, lateral, and the external of the plant LRR-RLK ectodomain superhelix. The signature of residues located − 5 to + 5 from N^+^ and N^−^ are shown in weblogos, where hydrophilic, neutral and hydrophobic residues are colored blue, green, and black, respectively. **g** and **h** The number of N^+^ and N^−^ at the N terminal, C terminal, or the middle area of the superhelix. LRRs located in the N terminal, middle, and C terminal LRRs were noted. N^+^ and N^−^ located in the internal side, lateral side, and backside of the N terminal, middle, and C terminal of the superhelix were calculated

Plant LRR-RLKs in SG_vii, SG_x_2*, SG_xi, and SG_xii had fewer sequential insertions (Fig. 3c) and tended to form superhelical structures with more than 20 LRRs. Well-studied proteins of this type are mainly proved to be receptors responsible for ligand recognition, which plays important roles in plant development and responses to environmental stresses [16, 18, 32, 33]. Since the structures of these types of LRR-RLKs could be well predictable through sequences (Fig. 4a), the distribution pattern of N^+^ and N^−^ (N sites not in the NxS/T (x ≠ P)) located on the internal, lateral, and external side of the LRR stacks in these LRR-RLKs were further depicted. Since a great amount of N^−^ located in the highly conserved N sites on the 9th sites of the CS on the inner side of the superhelix, N^−^ at these locations were ruled out. For asparagine residues on the internal and external side of the superhelix, only 40% were N^+^; in contrast, 70% located on the lateral side were N^+^ (Fig. 7a and c). A high ratio of over 85% of N^+^ sites lay on the 5th, 8th, 10th and the 21st variable sites in the plant LRR consensus sequences (Fig. 7c; Fig. 4d, variable residues colored in red), where the average soluble accessibility scores of the − 2, − 1, and + 1 residues next to N^+^ were very low (Fig. 7b). By contrast, ~ 50% N^−^ located on the 5th, 8th, 10th, and 21st variable sites (Fig. 7f), and the average soluble accessibility scores of the − 2, − 1, and + 1 residues next to N^−^ were higher than those of N^+^ (Fig. 7b and d). Interestingly, continuous N^+^ or N^−^ sites could be observed on the internal and the lateral side rather than the external side (Fig. 7c and f). Moreover, in comparison with N^−^, N^+^ on the inner side of the superhelix tended to locate at the N or C terminal rather than in the middle, whereas N^+^ located on the backside preferred the N terminal and the middle (Fig. 7g and h).

## Discussion

In this study, based on the PSSM algorithm of 16-residue plant-specific LRR-HCS (“LxxLxLxxNxLstGxIP”), a Phyto-LRR prediction program was constructed (Fig. 1). In employment of this LRR-prediction tool, more than 55,000 LRRs were detected from ~ 4000 protein sequences containing LRR(s), TM and KD domain from 17 land plant species (Table 1) were stored in a database for further analysis (http://phytolrr.com/). Sequences containing signal peptides further underwent ML phylogenetic analyses, and 18 SGs were then classified (Fig. S1; Tab. S3). The results revealed that although ancient species contained a lower number of LRR-RLKs (Table 3, Fig. 2a), the distribution pattern of the sequences are similar among species (Fig. 2b). The LRR(s) arrangement pattern (Fig. 3), the residues in the ligand-binding areas (Figs. 4 and 5), and the asparagine resides for N-glycosylation (Figs. 6 and 7) were then analyzed.

The position-specific scoring matrix (PSSM) is derived from a set of aligned sequences, therefore, this perceptron algorithm strongly depends on the training dataset [34]. There are three PSSM based LRR motif prediction programs, LRRfinder, LRRsearch, and the Phyto-LRR program. The LRR-finder program is trained with a non-redundant dataset comprising publically available Toll-like receptors (TLRs) sequences, which are the “typical” LRR class structure [26]. The position-specific scoring matrix (PSSM) was created to represent the 11-residue (LxxLxLxxNxL) LRR highly conserved amino acid positional distributions; the LRRsearch program was trained by the same 11-residue LRR highly conserved sections of 421 NOD-like receptors (NLRs) [25]; and the Phyto-LRR prediction program was trained by the 16-residue [LxxLxLxxNxL(s/t)GxLP] plant-specific LRR highly conserved sections from 17 representative land plants. Due to the preferences of the training datasets, the LRRfinder performed well for Toll-like receptors (TLRs), LRRsearch performed much better in cytoplasmic NOD-like receptors (NLRs) [25, 26], and the Phyto-LRR identified LRR motifs in plant LRR-RLK proteins more efficiently (Tab. S1). In comparison with LRRsearch, the Phyto-LRR could detect plant LRRs with divergent sequences, this should be attributed to that the perceptron of Phyto-LRR was trained with over 4000 plant LRR highly conserved motifs (Tab. S7) and was adjusted with the Laplace smoothing algorithm. The Phyto-LRR prediction module is available by local users at http://github.com/phytolrr so that a bunch of sequences could be detected at one time to better facilitate researches. Moreover, the training dataset function was open for users to trained the program to adjust to their own LRR-containing protein families, therefore, they could use the module to predict and analyze their interested LRR-containing protein families with high efficiency.

Plant LRR-RLKs are important membrane-localized receptors sensing various ligands to regulate plant developmental processes. The classification of LRR-RLKs is usually based on the alignment of the KDs, because the alignment of the ECDs is too ambiguous for phylogenetic analysis. In this work, ~ 3000 LRR-RLK sequences from 17 represented land plant species were classified into 18 SGs based on the alignment of their KDs. The SGs in the ML tree agree with most of the reported classifications [2, 6, 7, 12, 25, 26], and the robustness of the tree is supported by 10 test trees (Tab. S3). Similar to previously reported findings [10], most of the SGs had a similar pattern of LRR distribution in the ECDs, except SG_x, which was apparently divided into two distinct LRR distribution patterns (Fig. 3a). These two clusters were then named as SG_x_1* and SG_x_2* based on further Neighbor Joining phylogenetic analysis of SG_x (Fig. 3b), which was in well accordance with the phylogenetic branches by Fischer et al. [7]. The distinct LRR arrangement of SG_x_1* and SG_x_2* indicated that they might be involved in distinct mechanisms when activating signal transductions [16] and support the previous hypothesis that fusion of the kinase domain with different extracellular structures led to the current land plant RLK gene family [2, 12]. Interestingly, although the SG_x were divided into two subclades based on Neighbor Joining, it was monophyletic according to the ML tree and the two subclades SG_x_1* and SG_x_2* were intersected in the ML topology (Fig. S1), therefore further phylogenetic analysis of SG_x would unveil more evolutional significance of this SG. Comparison of paralogous genes revealed many LRR-RLK SGs have a ω > 1 (dN/dS ratio, the non-synonymous/synonymous substitution rates) [2, 7], indicating a net acceleration of protein evolution [35]; these genes are mainly distributed in the ECDs [2, 36, 37], especially for those bolded and underlined residues in the Lx**x**L**x**L**xx**N segment [7], located on the inner side of the superhelices assemblies [16, 18]. Several well-documented LRR-RLKs, such as BRI1 [20, 38], PSKR [39], RGFR1 [40], HAE [30], TDR/PXY [41], FLS2 [42], and PEPR1 [43], have been crystallized in forms of receptor-ligand binding. When analyzing the residues at each LRR in their homologs, it could be observed that the seemingly variable residues are distinct among homolog subclades, but to some extent conserved within each clade (Fig. 5), especially for residues located the ligand-binding domain (red bars in Fig. 5). Some of these highly conserved residues in Fig. 5 have been reported to be essential for ligand recognition in the Arabidopsis homologs, such as the RxR motif in the SG_xi homolog clades [16], S437 and T342 in FLS2 clades [42], and G186, Y188, G210, Y234, D255, D303, S305, W353, D375, and S377 in PXY [41]. Others, such as D414 in FLS2 and D273 in EPER1 clades, are to some extent varied, although the residue at these sites in the Arabidopsis homologs interacted with ligands [42], which might result from the functional mechanism variation among homologs in different plant species. The roles of those that are highly conserved in homologs and are located in the receptor-ligand interaction regions remain obscure and require further investigation. Therefore, this type of LRR prediction together with the proper modeling of the 3D structure will favor the study of plant LRR-RLKs. For example, residues lying on the 7th sites in each LRR often showed Ser residues (Fig. 5), and continuous serine residues tend to appear in LRRs not involved in ligand binding. Intriguingly, three weak BRI1 mutations [*bri1–9*, *bri1–706* (S253F), and *bri1–235* (S156F)] were identified and have been proved to be structurally imperfect but functionally competent mutants [44–46]. Most recently, the author (2020) found that the serine residues at the 7th site in AtBRI1 played important roles in protein proper folding in ER, while those non-serine residues at the ligand-binding region along the 7th sites were crucial for AtBRI1 function [47]. Moreover, residues lying on the 5th and 7th sites in each LRR often showed DLS/NLS motifs (Fig. 5), and continuous DLS/NLS motifs tend to appear in LRRs not involved in ligand binding. According to the BRI1 PDB files (3RGZ), there were polar contacts between the D and S residues in each LRR. Moreover, the NLS would supply N-glycosylation sites, which are beneficial for protein folding. These findings indicated that the DLS or NLS motifs should contribute to the protein structural integrity, yet more need to be done to reveal the underlying mechanisms. Furthermore, the island domain, an insertion section between LRRs is believed to be of great functional importance in LRR-RLK ligand binding [16, 32, 33], therefore Phyto-LRR’s efficient detection of the island domain will also favor the LRR-RLK functional study.

In *Arabidopsis*, based on the NxS/T (x ≠ P) motif, approximately ~ 1200 out of 4000 secretory glycoproteins contain more than five canonical N-glycosylation sites [48]. The N-glycoproteomic studies from representative eukaryotes showed that approximately 45% of the identified proteins have more than one identified N-glycosylation sites [49, 50]. In total, 82% of Arabidopsis LRR-RLKs had more than five canonical N-glycosylation sites (NxS/T (x ≠ P) sequons). LRR-RLKs with multiple N-glycosylation modifications have been confirmed by crystalizing [20, 30, 38, 39, 41, 42] or proteome analysis [49]; however, due to the limits of the technologies, the results are still not comprehensive [51]. Since most of the NxS/T (x ≠ P) sequons tended to be modified with N-glycans according to the manually checked Arabidopsis proteome GFF file from Swiss-Prot (Tab. S4), the analysis of the potential N-sites might help with the understanding of the function of this modification. These heavy N-glycosylation modifications are crucial for LRR protein structure and biological functions [22, 23, 52, 53]. In this work, N sites in the NxS/T (x ≠ P) sequons (N^+^ in this work) tended to localized at the 5th, 8th, 10th, and 21st variable sites in the plant LRR consensus sequences (Fig. 7c; Fig. 4d, variable residues colored in red). Moreover, the average soluble accessibility pattern of resides − 5 to + 5 next to the N sites was similar to that of N^+^ and N^−^, although the − 2, − 1, and + 1 residues were more hydrophobic for N^+^ than N^−^, indicating that N-glycosylation modification in LRR-RLKs tended to cover the local hydrophobic patches [55], and the deletion of one, might, to some extent, not cause dramatic destruction of the receptor structures [19, 53, 54] (Fig. 7b and e). Therefore, for individual LRR-RLKs/RLPs, the contributions of the N-glycans at different sites might not be identical: some sites are seemingly erasable without conferring any impacts on protein folding and bioactivity, and some could play critical roles for protein abundance and ligand recognition independently [19, 21]. More informative mechanisms underlying site-specificity of the N-glycosylation modifications should be interpreted based on the analysis of the crystal structures or the homolog modelling structures [55].

## Conclusion

Based on the “Phyto-LRR prediction”, an effective program for predicting the LRR segments in plant LRR-RLKs, the plant LRR-RLKs ECDs were comprehensively analyzed, revealing important characteristics of the residues in LRR motifs. This LRR prediction program and the ECD database will benefit the functional research of plant LRR-RLKs.

## Methods

### Studied genomes

In total, genomes from 17 representative land plants were analyzed (Table 1), including angiosperms (4 monocots [sub] species and 10 dicots species), liverwort, moss and spikemoss: *Phoenix dactylifera*, *Oryza sativa ssp. japonica*, *Oryza sativa ssp. indica*, *Brachypodium distachyon*, *Zea mays*, *Solanum tuberosum*, *Solanum lycopersicum*, *Arabidopsis thaliana*, *Arabidopsis lyrata*, *Brassica rapa*, *Populus trichocarpa*, *Glycine max*, *Medicago truncatula*, *Amborella trichopoda*, *Marchantia polomorpha*, *Physcomitrella patens*, and *Selaginella moellendorffii*. Throughout this article, the species were referred using five-digit identifiers as shown in Table 1. Details on genome versions can be found in Tab. S5.

### The extraction of the potential LRR-RLK protein sequences

Protein sequences containing both intact (i.e. non-degenerated) LRR(s) and a KD were extracted by running the hmmsearch (HMMER 3.2.1) program as described previously [56]. The TMs were predicted using TMHMM http://www.cbs.dtu.dk/services/TMHMM/ websites hosted at the Center for Biological Sequence Analysis, Technical University of Denmark [57]. Protein sequences containing LRRs, a TM and a KD were obtained and those encoded with the same gene ID were further filtered by picking up the longest sequences and sequences with unexpected characters were also removed. The ECDs and KDs of the protein sequences were then extracted, respectively, according to the ClustalW alignment in Mega 5.0 with default argument settings [58]. The obtained ECD sequences and the KD sequences were then checked by similar hmmsearch program for LRR and KD search, respectively (E value cut-off < 1). The non-redundant sequences (3987 sequences), which had LRR(s) in the N termini side, TM and a KD in the C termini, were taken as LRR-RLKs and were used for LRR motif prediction by Phyto-LRR prediction program and stored in the Phyto-LRR database. 2999 out of 3987 sequences were obtained after filtering with SignalP 5.0 [57] at http://www.cbs.dtu.dk/services/SignalP/, which were taken for further phylogenetic analysis and sequential assessment in the current article.

### The Phyto-LRR prediction program

The Phyto-LRR prediction program was constructed based on the PSSM algorithm as described previously [25] with some optimizations (Fig. 1). To avoid the zero-probability problem, the Laplace smoothing algorithm was used when the basic position frequency matrix (PFM) convert to the position probability matrix (PPM). The overlapping LRR motifs were discarded by selecting the non-overlapping LRR group with the highest score. There were two steps of training to create the PSSM weight matrix. Firstly, a total of 98 Arabidopsis LRR-RLK protein sequences containing more than 4 LRRs were chosen. The LRR motifs were extracted according to the annotation in UniProt at https://www.uniprot.org/ and the NCBI at https://www.ncbi.nlm.nih.gov with manual verification. ClustalW alignment was carried out in MEGA 5.0 to get a snapshot of the highly conserved sequence segments [58]. A total of 1467 16-residue segments, “LxxLxLxxNxLs/tGxIP”, were used for the first-round training (Tab. S6). Secondly, 10% of the 2999 sequences were randomly selected and 8 Arabidopsis LRR-LRKs with crystal structures were also added. The LRR motifs were then predicted from these sequences by the Phyto-LRR prediction program with manual verification. The 16-residue segments were used for the second-round training to adjust the PSSM weight matrix (Tab. S7).

### Construction of the Phyto-LRR database

The plant LRR-RLK database (Phyto-LRR database) was created using MySQL. A total of 3987 non-redundant ECD sequences of plant LRR proteins from 17 plant species (Table 1) were inserted into the database and each entry was updated with additional information such as the gene ID and the ECD length. The LRRs were identified by the Phyto-LRR prediction program, and the results were manually checked before integrated into the database. The deleted LRR motifs were shown in the database (http://phytolrr.com), and those manually added into the database were listed in Tab. S2A. The LRR motif candidates were listed in Tab. S2B. The database was also integrated with the prediction of the sequence second structures using the SSpro in the SCRATCH-1D suite [31]. Potential canonical N-glycosylation sites [Asn-x-Ser/Thr (x ≠ Pro)] were also included in the database.

### Sequences clustering, phylogeny, and analyses

In the present article, 2999 sequences containing signal peptide, LRRs, TM and KD were used for further phylogenetic, sequential and N-glycosylation motifs analysis. The SGs were classified using the KDs by global phylogenetic analysis (Table. 3; Fig. S1; Tab. S3). The KD sequences were aligned and cleaned with MAFFT (v7.245) [59] and trimAl [60] as described by Fischer et al. (2016) [8]. A maximum likelihood (ML) phylogenic tree was inferred using IQtree with autodetected models (JTT + F + G4 model) [61, 62]. Commands to generate the ML tree are available at http://github.com/phytolrr. SGs were defined manually using the Arabidopsis genes as a reference [2, 9]. The monophyletic type of each SG was further confirmed by ten ML trees in IQtrees from ten subsets of about 300 sequences, which were created by picking approximately 10% sequences randomly from each SG noted in the global phylogenetic tree (Tab. S3). The alignments of the KDs in SG_x were performed using ClustalW and MUSCLE programs in Mega 7 [63]. The phylogenetic tree was constructed using the Neighbor Joining (NJ) method in Mega 7. A total of 1000 bootstrap replications were performed to test the robustness of internal branches. The number of LRR motifs of each sequence and the distribution of the asparagine (N) sites in the potential N-glycosylation sties (N^+^) were then determined based on the Phyto-LRR database. The soluble accessibility was predicted by ACCpro20 programs in the SCRATCH-1D suite [31] and the average ACC20 values of the residues around N^+^ were also examined (Tab. S8). The data was then analyzed and showed using ggplot2 package in R [64]. Codes to generate Tab. S8 are available at http://github.com/phytolrr.

### The residue analysis at the inner side of the LRR-RLK ECDs

The Arabidopsis BRI1 (AT4g39400), PEPR1 (At1g73080), FLS2 (At5g46330), HAE (At4g28490), TDR/PXY (At5g61480), PSKR1 (At2g02220), and RGFR1 (At4g26540) protein sequences were used as queries to perform BLASTP search for their own homologous sequences in these 2999 LRR-RLK sequences (Table 3; Fig. S1; Tab. S3). For each group of sequences, protein sequences of the top 300 hits were selected and ten hits (or all of the hits if the total hits were less than 10) in each species were then chosen (Tab. S9). The KDs were aligned using MAFFT (v7.245) [59] with auto settings. The alignments were cleaned using TrimAl [60] with settings to only remove sites with more than 80% of gaps and the ML tree was inferred in IQtree with auto-detected models [61, 62]. Sequences from the BRI1, PEPR1, FLS2, HAE, TDR/PXY, PSKR1, and RGFR1 subclades were extracted for further residue analysis (Fig. S2). For each clade, full sequences of the homologs were aligned, and the LRR motifs of each sequence were denoted using the LRR offsets of the query sequences in the Phyto-LRR database as indicators. The LRRs of the homologs denoted in this way were highly identical with those in the Phyto-LRR dataset (> 95%), therefore the aligned LRRs were then slightly manually modified with the LRRs denoted in the database. The residues of each LRR segment located on the superhelical inner side (the LxxLxLxxN segment) were extracted and the residue logos of each site on each LRR among the homolog sequences (Fig. S3) [47] were showed in weblogo [65]. Codes are available at http://github.com/phytolrr.

## Supplementary Information

**Additional file 1: Figure S1.** The maximum-likelihood phylogenic analyses of ~ 3000 LRR-RLK sequences from 17 land plants in Table 1. **Figure S2.** The phylogenic analyses of LRR-RLK homologs. **Figure S3.** The procedure to create the residue logo of Fig. 5

**Additional file 2: Table S1.** The comparison of LRR predicting programs for predicting plant LRR-RLKs based on PSSM algorithm

**Additional file 3: Table S2.** LRR motifs manually added to the Phyto-LRR database

**Additional file 4: Table S3.** LRR-RLK members in each SG, refined neighbor-joining (NJ) tree of SG_x, and ten test sets of the phylogenetic tree

**Additional file 5: Table S4.** N-glycosylation sites annotated in the Arabidopsis proteome GFF file from Swiss-Prot

**Additional file 6: Table S5.** Information on the protein sequence datasets

**Additional file 7: Table S6.** Training dataset 1 for Phyto-LRR predict program

**Additional file 8: Table S7.** Training dataset 2 for Phyto-LRR predict program

**Additional file 9: Table S8.** The distribution pattern of LRR motifs and potential N-glycosylation sites of each LRR-RLK member in different SGs

**Additional file 10: Table S9.** Protein sequences blast from 17 plant genomes LRR-RLKs using Arabidopsis protein sequences as a query

## Data Availability

The “Phyto-LRR prediction” program can be used both online and offline. To predict LRR motifs online, please visit https://phytolrr.com/findlrr. The program is also provided as a PyPI module which could be installed by the command “pip install predict-phytolrr”. All source code can be found at “https://github.com/phytolrr/phytolrr“and “https://github.com/phytolrr/predict-phytolrr“. The database and generation command can be found at “https://github.com/phytolrr/database.

## Abbreviations

LRR-RLKs: Leucine-rich-repeat receptor-like kinases
ECDs: Extracellular domains
N-glycosylation: Asparagine-linked glycosylation
TM: Trans-membrane domain
KD: Kinase domain
SGs: Subgroups
CS: Consensus sequences
HCS: Highly conserved segment
PSSM: Position-specific scoring matrix algorithm
