# Supplementary material for: Identification and characterization of the LRR repeats in plant LRR-RLKs
Source: BMC Mol Cell Biol. 2021 Jan 28;22:9. doi: 10.1186/s12860-021-00344-y (PMC7841916; doi:10.1186/s12860-021-00344-y)
Supplement: Supplementary file 1 — Additional file 1: Figure S1. The maximum-likelihood phylogenic analyses of ~ 3000 LRR-RLK sequences from 17 land plants in Table 1. Figure S2. The phylogenic analyses of LRR-RLK homologs. Figure S3. The procedure to create the residue logo of Fig. 5 [file 12860_2021_344_MOESM1_ESM.pdf]

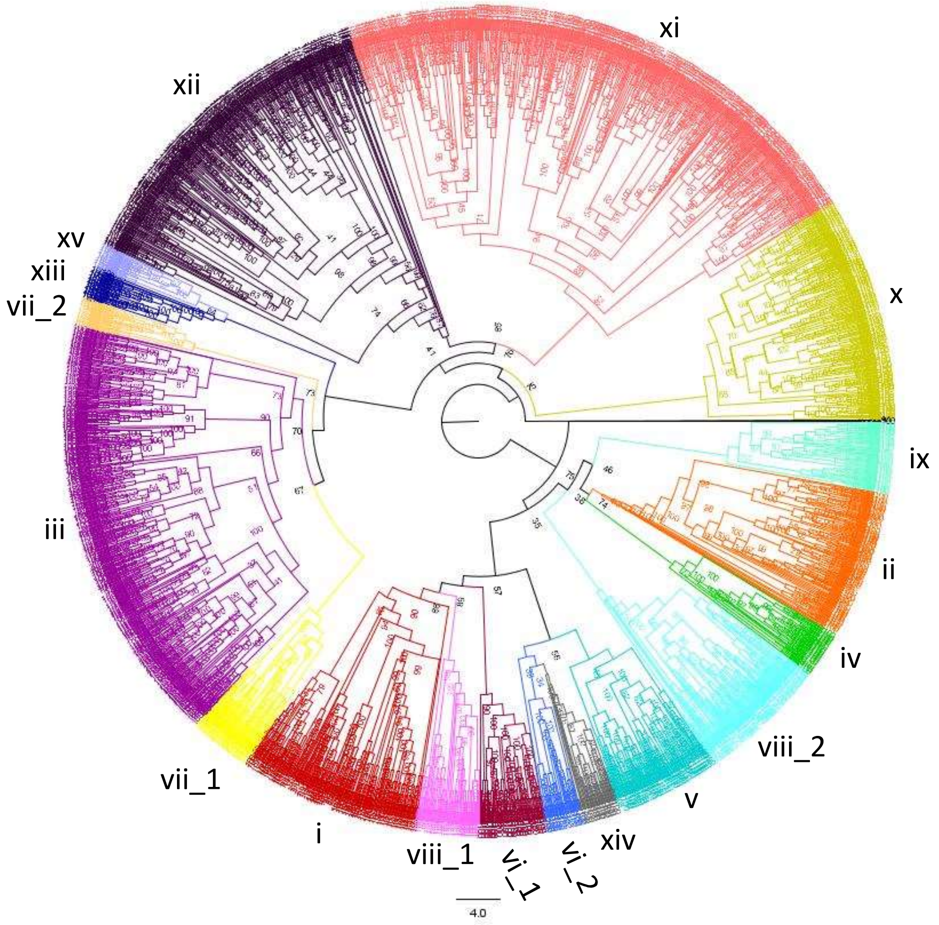

Figure S1 The maximum likelihood phylogenic analyses of ~3000 LRR-RLK sequences form 17 land plants in **Tab. 3**. The bar indicated a mutation rate of 4.0 substitutions per site. Bootstrap values were shown near the nodes. The summarization of the SGs were listed in **Tab. 3**. Sequences in each SG were listed in **Tab. S3**.

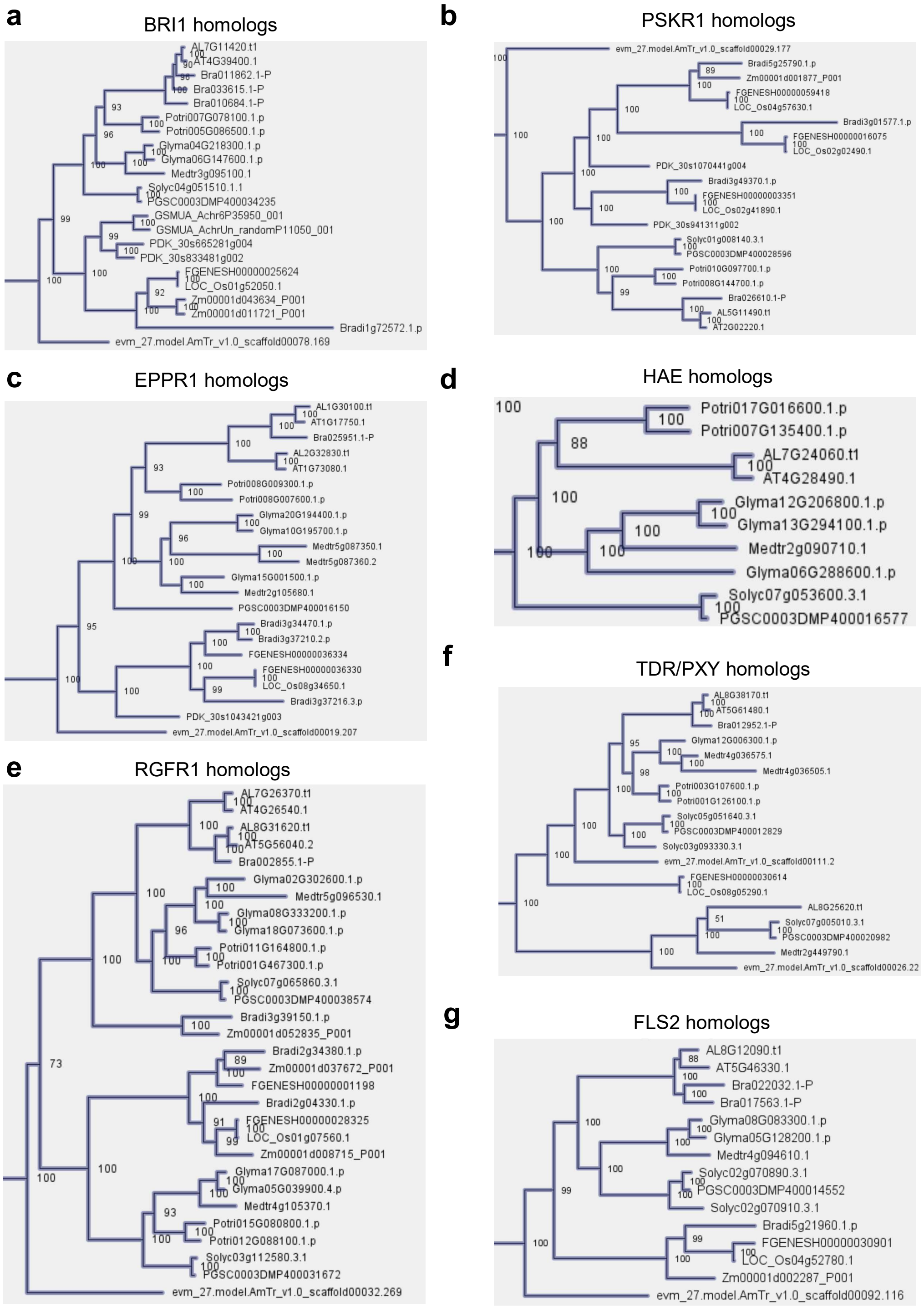

Figure S2 The phylogenic analyses of in BRI1 (a), PSKR1 (b), PEPR1 (c), HAE (d), RGFR1 (e), TDR/PXY (f) and FLS2 (g) homologs from 17 land plants in **Tab. 1**. The Arabidopsis protein sequences was used as a queries to perform BLASTP search for homologous sequences in 17 species genomes. The KDs of protein sequences of top 10 hits in each genome was used for the construction of the maximum likelihood (ML) phylogenetic tree in IQtree (see Methods). Subclades of the close homologs of the query sequences was shown. Bootstrap values of 1000 replicates were shown near the nodes

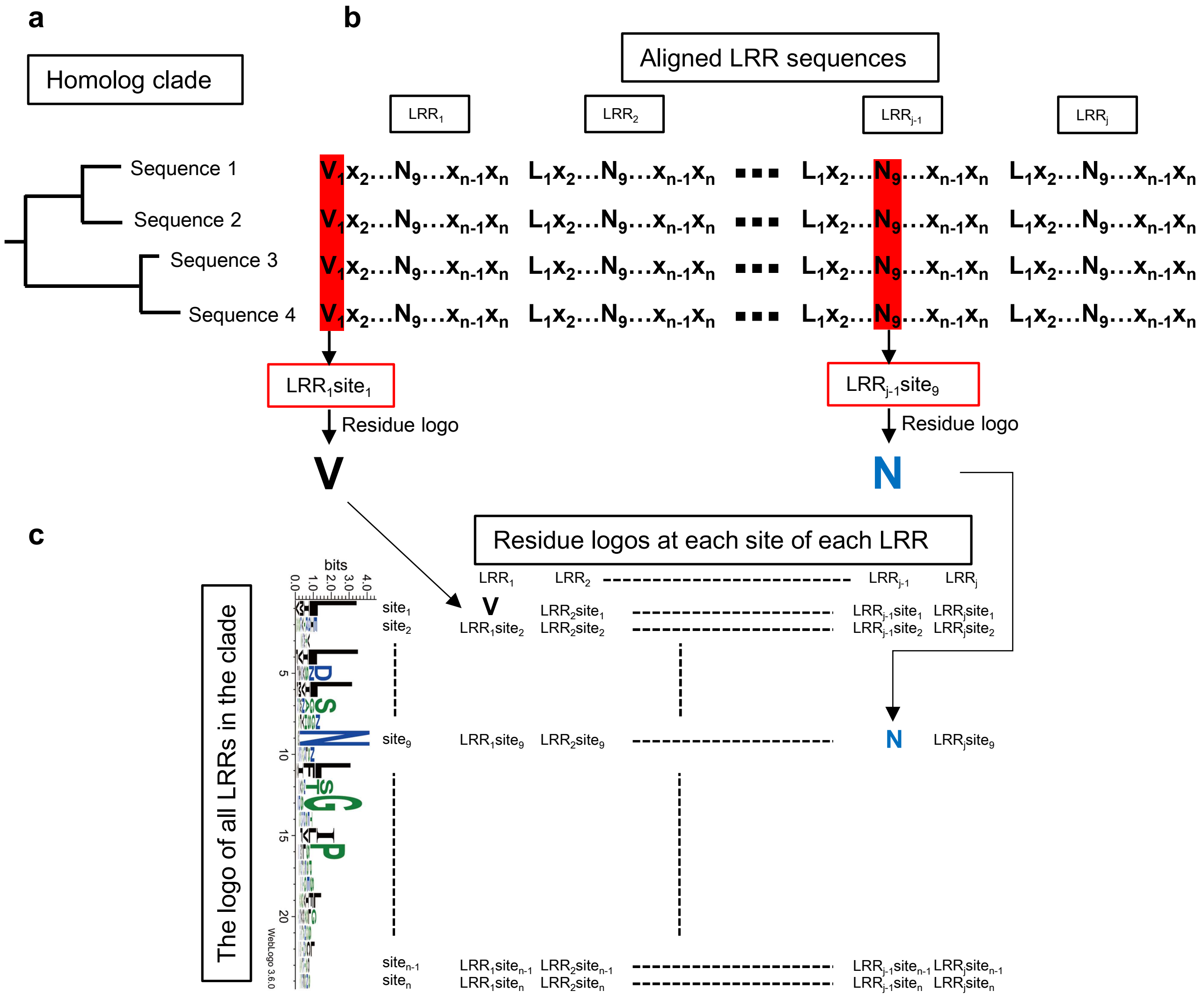

Figure S3 The procedure to create the residue logo of Fig.5. (a) Construct homolog sequences. (b) The sequences were well aligned, and the LRR motifs were confirmed based on the alignment and the Pytho-LRR database. (c) The residue logo of the LRRs of the clade and the logo of the residue at each site of each LRR were created and displayed using weblogo (see Methods).
