# Supplementary material for: Identification and characterization of the LRR repeats in plant LRR-RLKs
Source: BMC Mol Cell Biol. 2021 Jan 28;22:9. doi: 10.1186/s12860-021-00344-y (PMC7841916; doi:10.1186/s12860-021-00344-y)
Supplement: Supplementary file 2 — Additional file 2: Table S1. The comparison of LRR predicting programs for predicting plant LRR-RLKs based on PSSM algorithm [file 12860_2021_344_MOESM2_ESM.docx]

**Table S1** The comparison of LRR predicting programs for predicting plant LRR-RLKs based on PSSM algorithm. Two LRR-RLK sequences from each of the 17 plant species were randomly picked and the LRR motifs of the ECDs were then detected by the phyto-LRR prediction program, the LRRfinder program and the LRRsearche program, respectively.

| **Protein IDs** | **Species** | **phyroLRR-prediction** | **LRRfinder** | **LRRsearch** |
| --- | --- | --- | --- | --- |
| AL1G29470.t1 | Arabidopsis lyrata | 26 | 21 | 26 |
| AL1G50610.t1 | Arabidopsis lyrata | 27 | 24 | 25 |
| AT1G12460.1 | Arabidopsis thaliana | 17 | 15 | 17 |
| AT1G53430.1 | Arabidopsis thaliana | 10 | 7 | 9 |
| Bradi1g17135.1.p | Brachypodium distachyon | 23 | 20 | 22 |
| Bradi1g10950.3.p* | Brachypodium distachyon | 5 | 4 | 5 |
| Mapoly0013s0136.1.p | Marchantia polymorpha | 19 | 17 | 18 |
| Mapoly0003s0013.1.p | Marchantia polymorpha | 15 | 11 | 14 |
| evm_27.model.AmTr_v1.0_scaffold00005.159 | Amborella trichopoda | 9 | 6 | 7 |
| evm_27.model.AmTr_v1.0_scaffold00010.292 | Amborella trichopoda | 17 | 15 | 17 |
| PDK_30s65509272g001* | Phoenix dactylifera | 6 | 6 | 6 |
| PDK_30s6550932g001 | Phoenix dactylifera | 20 | 16 | 19 |
| Pp3c10_16000V3.2 | Physcomitrella patens | 9 | 8 | 8 |
| Pp3c12_14020V3.1 | Physcomitrella patens | 9 | 7 | 8 |
| Potri001G117800.1.p | Populus trichocarpa | 7 | 6 | 6 |
| Potri001G472900.1.p | Populus trichocarpa | 26 | 23 | 26 |
| Glyma01G028700.1.p* | Glycine max | 5 | 5 | 5 |
| Glyma01G125200.1.p | Glycine max | 8 | 6 | 7 |
| Medtr1g022265.1 | Medicago truncatula | 10 | 8 | 10 |
| Medtr2g016500.1 | Medicago truncatula | 5 | 4 | 4 |
| FGENESH00000000037 | Oryza sativa ssp. Indica | 11 | 7 | 9 |
| FGENESH00000008030 | Oryza sativa ssp. Indica | 8 | 7 | 7 |
| LOC_Os01g42294.1 | Oryza sativa ssp. Japonica | 6 | 5 | 5 |
| LOC_Os09g25540.1 | Oryza sativa ssp. Japonica | 22 | 19 | 22 |
| EFJ38333 | Selaginella moellendorffii | 6 | 5 | 6 |
| EFJ38110 | Selaginella moellendorffii | 22 | 19 | 22 |
| Solyc08g075590.2.1* | Solanum lycopersicum | 26 | 23 | 26 |
| Solyc05g052350.3.1 | Solanum lycopersicum | 13 | 11 | 12 |
| PGSC0003DMP400017360 | Solanum tuberosum | 6 | 5 | 6 |
| PGSC0003DMP400029218* | Solanum tuberosum | 7 | 7 | 7 |
| Zm00001d013415_P001 | Zea mays | 13 | 7 | 10 |
| Zm00001d021883_P001 | Zea mays | 5 | 4 | 4 |
| Bra008427.1P | Brassica rapa | 13 | 9 | 16 |
| Bra021551.1P | Brassica rapa | 7 | 6 | 7 |

*Sequence IDs with asterisks indicated that some LRRs were predicted with different offsets in different programs although they were detected with the same number of the total LRR motifs.
